# Computational Pupillometry and the Pupil Reactivity (PuRe) Score in Neurocritical Care: Prospects for High-Accuracy, Lighting-Invariant Neuromonitoring

**DOI:** 10.7759/cureus.92537

**Published:** 2025-09-17

**Authors:** Patryk Szczęśniewski, Małgorzata Dudzic, Karol Badowski, Michał Świątek, Martyna Płatnikow, Jakub Klawiter, Wiktor Olszewski, Marta Kowalow, Zuzanna Paryzek, Kacper Pawlak, Hugo Chrost, Michal Wlodarski, Marek Dziubiński, Radosław Chrapkiewicz, Artur Drużdż, Bartosz Sokół

**Affiliations:** 1 Department of Neurosurgery, Poznan University of Medical Sciences, Poznan, POL; 2 Department of Neurosurgery, Joseph Strus Municipal Hospital, Poznan, POL; 3 Research and Development, Solvemed Inc., Lewes, USA; 4 Department of Neurosurgery, Medical University of Warsaw, Warsaw, POL; 5 Ophthalmology, University College London Institute of Ophthalmology, London, GBR

**Keywords:** artificial intelligence, neurocritical care, neuromonitoring, pupillometry, pupil reactivity, pupil reactivity (pure)

## Abstract

Introduction

An evaluation of computational pupillometry, implemented through the pupil reactivity (PuRe) pupillometer smartphone-based device, was prospectively carried out in a neuro-ICU cohort to make a lighting-invariant assessment of pupil reactivity and its clinical utility in critical care.

Methods

This prospective single-center observational pilot study was conducted in the neuro-ICU of a municipal hospital in Poznań, Poland, in 2024. Adult patients admitted with acute neurological conditions such as hemorrhagic stroke, traumatic brain injury, brain tumor, hydrocephalus, or intracranial hypertension were monitored under varying ambient light conditions. Pupillometry measurements were collected using the PuRe pupillometer, a smartphone-based device that computes standard pupillary light reflex parameters and the lighting-invariant Pupil Reactivity (PuRe) Score. Neurological status was assessed via the Glasgow Coma Scale, and survival outcomes were recorded at discharge. Statistical analyses included comparisons across light conditions using non-parametric tests, correlations with clinical variables via Spearman's correlation, and receiver operating characteristic analysis for predictive thresholds.

Results

Twelve patients, with a median age of 59 years, contributed 1,331 measurements, at 44 measurements per patient, under ambient light ranging from 4 to 1,200 lux. Standard pupillary light reflex parameters showed high light dependence with variations up to 133% (p<0.001), whereas PuRe Scores remained stable with medians of 2.64 in dim light versus 2.42 in bright light (p=0.449; r=-0.026). PuRe Scores correlated strongly with neurological status (Spearman ρ=0.746, p<0.001). At a threshold below 3.0, the PuRe Score identified severe Glasgow Coma Scale scores (≤8) with 84.3% sensitivity, 90.2% specificity, 86% accuracy, and an area under the curve of 0.940. Non-survivors exhibited markedly lower median PuRe Scores of 0.00 compared to 2.82 in survivors (p<0.001), with 91.7% of non-survivor recordings below the 3.0 threshold. Frame-level analysis demonstrated pupil diameter accuracy of ±0.025 mm.

Conclusion

Lighting-invariant, artificial intelligence (AI)-driven pupillometry provides a precise, clinically meaningful assessment of pupil reactivity, enabling objective bedside neuromonitoring without strict light control and supporting early detection of severe injury and poor outcome.

## Introduction

A timely and objective neurological assessment is essential for effective patient management across most care delivery settings - from emergency departments to neurocritical care units (neuro‑ICUs). While many monitoring tools - such as intracranial pressure (ICP) monitors - provide valuable data, they are often limited by risks and can be resource‑intensive [1]. The pupillary light reflex (PLR) serves as a non‑invasive neurological marker, driven by retinal input to the mid‑brain olivary pretectal nucleus but is traditionally assessed subjectively [2]. Quantitative pupillometry, particularly infrared (IR)‑based systems [2,3], improves objectivity but remains confounded by ambient lighting conditions [4,5] and limited by built‑in computational capacity, thus precluding the use of computationally demanding computer vision algorithms and advanced data analysis techniques.

In view of these limitations [4,5], we tested the next‑generation computational pupillometry method, implemented via pupil reactivity (PuRe) Pupillometer, a US Food and Drug Administration (FDA) software as a medical device (SaMD) application (Solvemed Inc.). This approach leverages the significant processing power of modern mobile computing platforms, such as iPhones (Apple Inc., Cupertino, CA, USA), with their multi‑core graphical processing units (GPUs) and neural processing units (NPUs) dubbed as Apple Neural Engine (ANE). This enables the real‑time application of advanced artificial intelligence (AI) algorithms and sophisticated multi‑frame integration techniques, such as multi‑frame super‑resolution (MFSR), temporal averaging, and parallax‑based artifact mitigation [6]. PuRe Pupillometer computes the Pupil Reactivity (PuRe) Score [7,8], a lighting‑invariant metric designed to quantify pupillary dynamics and to overcome the ambient light sensitivity that challenges traditional systems [2,3].

This article provides findings from a prospective, single‑center observational pilot study conducted in a neuro‑ICU setting in a wide range of ambient light conditions (4-1,200 lux) among diverse patients suffering from stroke, traumatic brain injury (TBI), brain tumor, hydrocephalus, or intracranial hypertension, with common neuro‑ICU sedatives and analgesics. PuRe Pupillometer’s measurement accuracy, lighting‑invariance of derived metrics, and ability to predict the severity of neurological state and the patient’s outcome have been tested. In this study, we aimed to: quantify the light-dependence of standard PLR metrics; test whether the PuRe score is stable across light levels; report frame-level pupil-diameter accuracy; assess the association between PuRe and Glasgow Coma Scale (GCS); and explore a prognostic signal for in-hospital survival.

## Materials and methods

Study design

This single-center prospective observational pilot study was conducted in December 2024 at the neurological intensive care unit of Joseph Strus Municipal Hospital (Poznań, Poland). The study adhered to the principles outlined in the Declaration of Helsinki and received prior approval from the research ethics committee of Poznan University of Medical Sciences, with protocol number 419/24 granted on June 27, 2024. The Strengthening the Reporting of Observational Studies in Epidemiology (STROBE) cohort reporting guidelines were applied to ensure transparent and comprehensive reporting, including explicit descriptions of the study design, setting, participants, variables, data sources and measurements, efforts to address potential sources of bias, study size, handling of quantitative variables, and statistical methods. Enrollment ran in a calendar month (December 2024), with bedside follow‑up continuing daily until ICU discharge or death.

Setting

The study took place in a tertiary-level neurological intensive care unit equipped for managing acute neurological conditions, where patients received standard clinical care including mechanical ventilation, sedation, hemodynamic support, and neurosurgical interventions as indicated. Ambient lighting varied naturally across the unit, ranging from 4 to 1,200 lux, reflecting real-world conditions without artificial control. Follow-up extended from admission until intensive care unit discharge or death, with serial assessments integrated into routine clinical workflows.

Participants

Eligible patients were adults aged 18 years or older admitted to the neurological intensive care unit with acute neurological diagnoses such as hemorrhagic stroke, traumatic brain injury, brain tumor, hydrocephalus, or intracranial hypertension. For the patients, inclusion in the study required the capacity for serial pupillometry assessments starting from admission day zero, without pre-existing conditions that would preclude valid measurements, such as severe ocular pathology unrelated to the neurological insult. Exclusion criteria included non-neurological primary admissions and an inability to perform pupillometry due to logistical or mechanical constraints. Patients were enrolled consecutively to minimize selection bias. Informed consent was obtained directly from conscious participants; for unconscious patients, consent was waived by the ethics committee, with proxy consent sought from legally authorized representatives as soon as feasible.

Variables

Primary variables encompassed pupillometry-derived metrics, including initial pupil diameter, constriction amplitude, percent constriction, constriction velocity, and the Pupil Reactivity (PuRe) Score. Secondary variables included Glasgow Coma Scale scores as a measure of neurological severity, with severe impairment defined as scores of 8 or less; medications potentially affecting pupillary reactivity or consciousness, such as fentanyl, propofol, and noradrenaline; and clinical outcomes like intensive care unit length of stay and survival status at discharge. Confounders such as age, sex, primary diagnosis, and ambient light levels were recorded to contextualize the findings.

Data sources and measurement

Clinical metadata were obtained prospectively through standardized daily neurological assessment forms completed by the multidisciplinary care team during routine rounds, drawing from electronic health records, bedside observations, laboratory results, and imaging reports. These forms captured the Glasgow Coma Scale scores assessed hourly from admission day zero, alongside descriptions of the neurological status, changes relative to the previous day or admission, interventions, and relevant medications. Quantitative pupillometry was performed using the PuRe Pupillometer, a smartphone-based software as a medical device operating on an iPhone platform. Operators used an on‑screen guidance to reach a 150‑mm working distance. The app logs the estimated distance per acquisition (range 142.6-158.8 mm; median 149.8 mm). Across all recordings, PuRe showed a negligible association with distance as reported in the Results section. Measurements were recorded hourly from admission day zero, involving five-second video recordings that included a one-second baseline period, a one-second flash stimulation from the device's integrated light source, and a three-second recovery phase, captured at 60 Hz frame rates. Processing occurred on-device utilizing the Apple Neural Engine for artificial intelligence-based pupil and iris segmentation, alongside the graphical processing unit (GPU) and central processing unit (CPU) for deterministic signal analysis, yielding immediate display of pupillometry parameters and the PuRe Score. The results were synced online with secure servers for subsequent aggregation. Ambient light has been measured on-device using methods explained in [7] and verified using the hardware light meter (Abatronic 8809A; Abatronic Sp. z o.o., Radom, Poland).

Bias

The potential sources of bias were addressed through consecutive enrollment of all eligible neuro-ICU admissions during the study window, thereby minimizing selection bias. No diagnosis- or prognosis-based preselection was applied, and predefined inclusion/exclusion criteria were consistently followed. To contextualize the results and mitigate residual confounding, ambient light levels, medications, and relevant clinical variables were documented prospectively. Standardized measurement protocols with device-specific quality checks minimized information bias.

Online quality control was an integral part of the measurement flow. Operator-induced motion (lateral or axial) was automatically detected by computer-vision models, which imposed acceptance criteria for eye visibility and distance. Blinking, eyelid edema, or partial occlusion were recognized through confidence metrics in the pupil/iris segmentation process. Corneal glare was not problematic, as the algorithms were trained on images containing reflections. Motion blur was rare due to the 60 Hz acquisition rate; when present, recordings with frames exceeding blur thresholds were reported. Anisocoria was not treated as an artifact but explicitly preserved as a clinical result. This flow ensured that unreliable recordings were immediately flagged and reported to the operator, prompting repetition under improved conditions.

Study size

As a pilot study focused on feasibility and preliminary insights into lighting-invariant pupillometry, no formal sample size calculation was performed. The cohort comprised 12 consecutively enrolled patients, yielding 1,331 pupillometry measurements to provide sufficient data for exploratory analyses and effect size estimation for future trials.

Quantitative variables

Quantitative variables, including pupillometry parameters and Glasgow Coma Scale scores, were treated as continuous measures, with summaries using medians and interquartile ranges (IQRs) to account for non-normal distributions where applicable. Ambient light was categorized as dim (less than 100 lux) or bright (100 lux or greater) for stratified analyses, while the PuRe Score was analyzed as a scalar metric ranging from 0 to 5.

Statistical methods

Descriptive statistics summarized cohort characteristics and pupillometry performance, with medians and IQRs for continuous variables. Group comparisons, such as lighting effects on parameters, employed non-parametric tests like the Mann-Whitney U test. Correlations between the PuRe score and clinical variables, including Glasgow Coma Scale scores, utilized Spearman's rank coefficient. Discrimination analyses for severe neurological impairment used receiver operating characteristic curves. All analyses were performed using Python version 3.11 (Python Software Foundation, Wilmington, DE, USA) with libraries including numpy, scipy, pandas, statsmodels, matplotlib, and seaborn. Unless stated otherwise, percentages are reported at the patient level; recording‑level summaries are explicitly labeled ‘per‑recording’.

## Results

Participants

The cohort comprised nine men (75%) and three women, with a median age of 59.0 years (interquartile range 50.5-66.2 years). The median intensive care unit length of stay was 11.0 days (interquartile range 5.8-17.2 days). The primary diagnoses included hemorrhagic stroke in four patients (33.3%), traumatic brain injury in three patients (25.0%), brain tumor in two patients (16.7%), hydrocephalus in two patients (16.7%), and intracranial hypertension in one patient (8.3%). A total of 1,331 pupillometry recordings were collected, with a median of 44.0 recordings per patient (IQR 24.0-166.5 recordings). Ambient illumination during recordings ranged from 4 to 1,200 lux, stratified into dim conditions (<100 lux; n=380 recordings; median 50.0 lux, IQR 33.0-78.0 lux) and bright conditions (≥100 lux; n=951 recordings; median 150.0 lux, IQR 130.0-220.0 lux). Concomitant medications potentially influencing pupillary reactivity included propofol in 17.0% of recordings and fentanyl in 15.8% of recordings, reflecting routine neurocritical care pharmacotherapy. The median Glasgow Coma Scale score across recordings was 3.0 (IQR 3.0-15.0), with 54.5% of patients presenting severe impairment (Glasgow Coma Scale ≤8). Cohort characteristics and recording contexts are summarized in Table 1.

**Table 1:** Baseline cohort and recording context (patients = 12; recordings = 1,331). Values are median (Q1, Q3) or n (%) at the patient level.

Variable	Value
Age, years	59.0 (50.5, 66.2)
Sex, male	9 (75.0%)
ICU length of stay, days	11.0 (5.8, 17.2)
Total recordings	1,331
Recordings per patient	44.0 (24.0, 166.5)
Diagnoses – hemorrhagic stroke	4 (33.3%)
Diagnoses – traumatic brain injury	3 (25.0%)
Diagnoses – brain tumor	2 (16.7%)
Diagnoses – hydrocephalus	2 (16.7%)
Diagnoses – intracranial hypertension	1 (8.3%)
In-hospital survivors	10 (83.3%)
In-hospital non-survivors	2 (16.7%)

Pupillometry measurement accuracy

**Figure 1:**
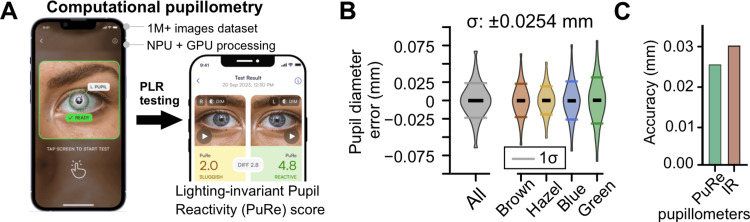
Computational pupillometry delivers high‑accuracy, lighting‑robust pupil measurements. (A) On‑device pipeline. A smartphone‑based computational pupillometer performs pupil light reflex (PLR) tests and processes the video frames locally: deep neural networks (trained on >10^6^ eye images) run on the phone’s neural processor to segment the pupil/iris; graphical processing unit/central processing unit (GPU/CPU) post‑processing yields frame‑wise pupil diameter, standard PLR metrics, and the lighting‑invariant Pupil Reactivity (PuRe) score. Image credit: Michal Wlodarski. (B) Per‑frame diameter accuracy across eye colors. Violin plots show the residual error (observed frame vs. low‑pass reference) for brown (n=482), hazel (n=365), blue (n=360), and green (n=124) irises. The error distribution is centered near zero with pooled single‑frame accuracy of ~±0.025 mm, demonstrating color‑independent precision. (C) Accuracy relative to infrared (IR) pupillometers. Summarized accuracy shows the computational system’s ~25‑µm pupil‑diameter precision, exceeding the ~30–100‑µm accuracy typically reported for infrared (IR) devices. Together with the Pupil Reactivity (PuRe) Score, this enables reliable, quantitative pupillometry on general‑purpose mobile hardware.

Computational pupillometry implemented via the PuRe Pupillometer on iPhone (Figure 1A) achieved high per-frame precision across iris colors, thanks to its on-device pipeline. This pipeline includes deep neural network algorithms trained on more than one million eye images and executed on the NPU, combined with deterministic mathematical algorithms running on the GPU and the central processing unit (CPU). Violin plots (Figure 1B) show residual frame‑wise pupil‑diameter errors versus a low‑pass reference centered near zero; pooled single‑frame accuracy was ±0.025 mm across brown (n=482 frames), hazel (n=365), blue (n=360), and green (n=124) irises. As summarized in Figure 1C, this ~25‑µm precision exceeds the ~30-100‑µm accuracy typically reported for infrared pupillometers [2,3]. This precision supports a reliable derivation of standard pupillary light‑reflex metrics and the lighting‑invariant PuRe score used in subsequent analyses.

Example case: tracking neurological change

**Figure 2:**
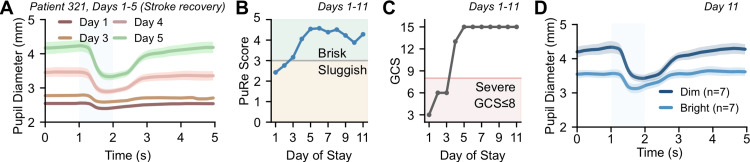
Example patient: non‑invasive neurological tracking during stroke recovery. (A) Pupillograms (Patient 321, days 1-5). Five day‑by‑day traces illustrate progressive strengthening of the pupil light reflex (PLR) response consistent with improving neurological status. Thick lines denote means with standard error of the mean (SEM) shaded area, vertical band mark the light-stimulation window. (B) PuRe score over the hospital course (days 1-11). Median daily PuRe values rise from the “sluggish” range to the “brisk” range, crossing the clinically relevant threshold of PuRe=3 that separates severe (GCS≤8) from non‑severe status. (C) Glasgow Coma Scale (GCS) over the same period. GCS improves from severe levels to 15 by day 5, resembling the PuRe trajectory and highlighting concordance between the quantitative pupillary marker and bedside neurological examination. (D) Pupillograms on the last day in dim vs bright light conditions.

Longitudinal tracking is illustrated using a representative patient (Figure 2). The median daily PuRe score increased from the sluggish into the brisk range over days 1-11, crossing the clinically relevant threshold of PuRe=3 that separates severe (GCS ≤ 8) from non‑severe status (Figure 2B), while the bedside examination improved to GCS=15 by day 5 (Figure 2C), indicating concordant trends between PuRe and neurological status. The same‑day traces acquired under dim and bright illumination show visibly different PLR time traces.

Influence of ambient light on PLR and lighting-invariance of PuRe

**Figure 3:**
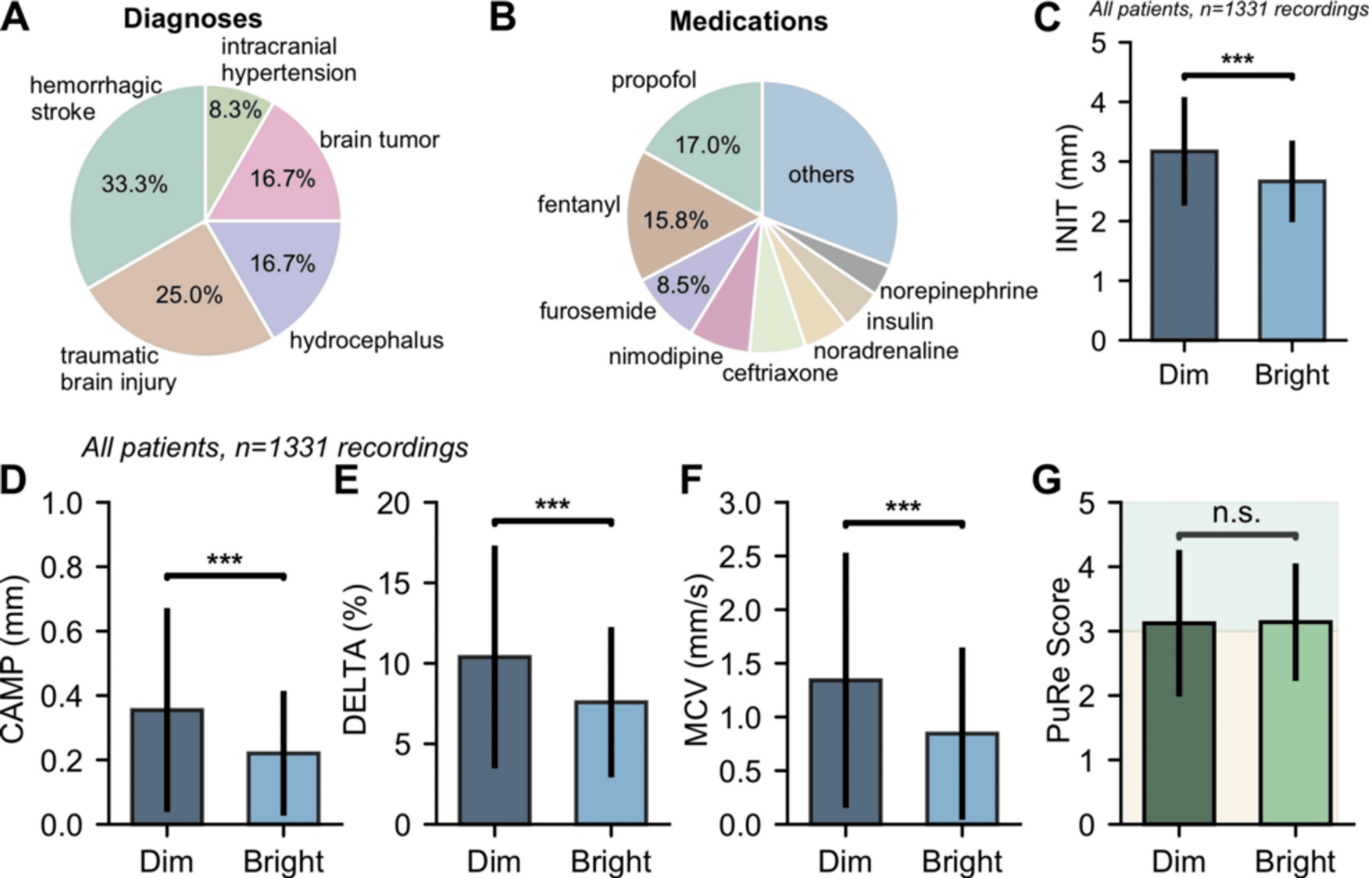
Study overview and light dependence of standard PLR metrics versus light invariance of PuRe. (A) Cohort diagnoses. Twelve neuro‑ICU patients contributed 1,331 recordings across diverse etiologies (i.e., hemorrhagic stroke, traumatic brain injury, hydrocephalus, brain tumor, intracranial hypertension). (B) Concomitant medications. Typical ICU treatments included propofol (17.0%), fentanyl (15.8%), and others; data reflect routine, real‑world practice. (C–F) Ambient‑light effects on conventional PLR parameters. Across ambient illumination spanning ~4–1,200 lux (categorized as dim <100 lux, n=380; bright ≥100 lux, n=951), standard PLR metrics differed significantly (p<0.001, Mann–Whitney U): INIT (initial diameter) ~41% larger in dim vs. bright (≈3.64 vs. 2.58 mm); CAMP (constriction amplitude) ~133% larger (≈0.32 vs. 0.14 mm); DELTA (percent constriction) ~95% larger (≈8.91% vs. 4.58%); MCV (max constriction velocity) ~82% larger (≈1.58 vs. 0.87 mm/s). These dependencies are also reflected by significant negative correlations with light level (e.g., INIT r=−0.433). (G) PuRe is lighting‑invariant. In contrast, PuRe showed no significant difference between dim and bright conditions (median 2.64 vs. 2.42; mean difference ~0.4%; p=0.449). This supports PuRe as a stable neuromonitoring metric independent of room lighting.

Standard pupillary light reflex parameters exhibited significant dependence on ambient illumination. Initial pupil diameter correlated negatively with light levels (r=−0.433, p<0.001), constriction amplitude (r=−0.235, p<0.001), and maximum constriction velocity (r=−0.268, p<0.001). Stratified by lighting level ranges, initial pupil diameter was 3.64 mm in dim conditions versus 2.58 mm in bright conditions (∼41% larger in dim condition, p<0.001), constriction amplitude 0.32 mm versus 0.14 mm (∼133% larger, p<0.001), percent constriction 8.91% versus 4.58% (∼95% larger, p<0.001), and maximum constriction velocity 1.58 mm/s versus 0.87 mm/s (∼82% larger, p<0.001). In contrast, after excluding non-reactive cases (PuRe Score=0), the PuRe Score showed no significant difference between dim (median 2.64, interquartile range 0.75-3.97) and bright conditions (median 2.42, IQR 0.00-3.45; p=0.449, Mann-Whitney U test), with a negligible correlation to distance of measurement (range 142.6-158.8 mm, median: 149.8 mm), PuRe-distance correlation: r=−0.026, p>0.05). These effects are detailed in Table 2 and visualized in Figure 3C-G, while patients' diagnoses and medications, potential confounders, are summarized in Figure 3A,B.

**Table 2:** PLR parameters and PuRe by ambient light (per-recording). Groups: Dim <100 lux (n=380) vs Bright ≥100 lux (n=951). INIT=initial diameter; CAMP=constriction amplitude; DELTA=percent constriction; MCV=maximum constriction velocity; PuRe=Pupil Reactivity (lighting-invariant). Mann-Whitney U tests (two-sided). PuRe medians/IQR reflect the analysis set used for lighting-invariance (non-reactive cases excluded by design).

Measure	Dim (<100 lux)	Bright (≥100 lux)	p-value
INIT, mm	3.64	2.58	<0.001
CAMP, mm	0.32	0.14	<0.001
DELTA, %	8.91	4.58	<0.001
MCV, mm/s	1.58	0.87	<0.001
PuRe score (0-5)	2.64 (0.75, 3.97)	2.42 (0.00, 3.45)	0.449

Association with neurological status

**Figure 4:**
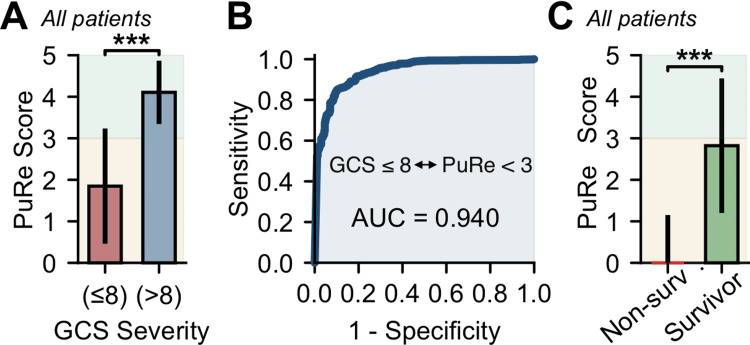
PuRe discriminates neurological severity and outcome. (A) Severity stratification by Glasgow Coma Scale (GCS). Recordings from severe (GCS≤8; n=896) versus non‑severe (GCS>8; n=373) states show markedly lower PuRe in the severe group (median ~1.85 vs. ~4.11; p<0.001). Error bars show the standard deviation (SD). (B) Receiver‑operating characteristic (ROC) for classifying severe status. A threshold Pupil Reactivity (PuRe) Score  ≤3.0 identifies GCS≤8 with AUC (area under the curve)=0.940, sensitivity 84.3%, and specificity 90.2% (overall accuracy 86.0%). Shaded regions illustrate the operating range. (C) Association with survival. PuRe is substantially lower in recordings from non‑survivors (n=248) than survivors (n=1,083) (p<0.001); most non‑survivor measurements fall below PuRe 3.0, highlighting potential prognostic utility. Panel C summarizes PuRe by survival status across all recordings (descriptive); the survivor set still includes many severe-state (GCS≤8) recordings. The PuRe≤3.0 threshold is used in Panel B for per-recording GCS severity classification, where GCS>8 recordings show PuRe ≈4.11 in keeping with the threshold (Table 3). Study‑wide notes (applies to all panels as relevant): Twelve adults in a single‑center neuro‑ICU contributed 1,331 measurements across variable ambient lighting. Statistics are Mann-Whitney U.

The PuRe Score correlated strongly with Glasgow Coma Scale scores (Spearman ρ=0.746, p<0.001). Recordings from severe neurological states (Glasgow Coma Scale ≤8; n=896) had a mean PuRe Score of 1.85±0.074 (standard error of the mean) compared to 4.11±0.050 in non-severe states (n=373; p<0.001), excluding recordings without time-matching of PuRe-GCS values. For detecting severe impairment, a threshold of PuRe Score ≤3.0 yielded an area under the receiver operating characteristic curve of 0.940 (95% confidence interval 0.92-0.96), with sensitivity 84.3%, specificity 90.2%, accuracy 86.0%, positive predictive value 95.4%, and negative predictive value 70.3%. These associations and operating characteristics are presented in Table 3 and Figure 4A,B. Figure 4B presents a per-recording classification analysis by GCS category, where recordings with GCS>8 have substantially higher PuRe (≈4.11) than those with GCS≤8 (≈1.85), consistent with the PuRe≤3.0 threshold for severe status (Table 3).

**Table 3:** Diagnostic performance of PuRe for severe neurological status (per-recording) Classification performance metrics for PuRe predicting severe neurological status (GCS≤8), per-recording. AUC‑ROC: Area under the receiver operating characteristic curve. 95% CI: 95% confidence interval. PPV: positive predictive value. NPV: negative predictive value.

Metric	Value
Target condition	GCS≤8
Dataset (per-recording)	n=1,269
Decision threshold	PuRe≤3.0
AUC-ROC	0.940
95% CI (lower)	0.92
95% CI (upper)	0.96
Sensitivity, %	84.3
Specificity, %	90.2
Accuracy, %	86.0
PPV, %	95.4
NPV, %	70.3

Prognostic value for outcomes

Non-survivors (n=248 recordings) exhibited a median PuRe Score of 0.00 (IQR 0.00-1.98) compared to 2.82 in survivors (n=1,083 recordings; IQR 1.61-3.83; p<0.001). Notably, 91.7% of non-survivor recordings fell below the 3.0 threshold (Table 3, Figure 4C). This survival comparison in Figure 4C is descriptive and aggregates all recordings across each patient’s ICU course; in our cohort, the survivor group still contained a large share of recordings acquired during severe neurological states (GCS≤8). Consequently, the survivor mean/median PuRe can lie below 3.0 without contradicting the GCS-based per-recording classification in Figure 4B; indeed, non-severe recordings (GCS>8) exhibit PuRe ≈4.11 (Table 3).

## Discussion

This study demonstrates that computational pupillometry can represent a paradigm shift in neurological assessment and monitoring. The PuRe Score’s lighting invariance addresses a critical limitation of traditional pupillometry [4,5], enabling reliable measurements in variable ambient lighting conditions, a common challenge in clinical environments. For example, in an ICU, ambient light levels can vary by up to five orders of magnitude.

The system’s pupil diameter accuracy of 25 μm already exceeds that of conventional infrared pupillometers (30-100 μm) [2,3], thanks to the combination of high‑resolution iPhone cameras combined with AI‑driven processing that can exploit rather than mitigate environmental variability using image scene micro‑movements and variable lighting as sources of enhanced measurement through multi‑frame integration [6]. While the current implementation uses iPhone hardware to standardize optics, light-emitting diode (LED) stimulus, and on-device neural processing (ANE), in the future this software-as-a-medical device may be ported to other smartphones with compatible optics, sensors, neural engines, and operating systems.

The strong correlation of PuRe with GCS and accurate discrimination of severe neurological impairment (AUC=0.940, misclassification 14%) demonstrates its clinical utility. Its potential prognostic utility is suggested by the larger difference in PuRe Scores between survivors and non‑survivors, with 91.7% of non‑survivor recordings falling below the critical threshold of PuRe=3.0. This suggests potential for early identification of patients at the highest risk.

Quantitative pupillometry (QP) is rapidly supplanting subjective penlight exams by delivering objective, reproducible pupillary metrics across multiple sites. Secondary analyses of multicenter cohorts show that neurological pupillometry monitoring correlates closely with intracranial hypertension in patients with acute brain injury [9], and automated pupillometry can identify the absence of intracranial pressure elevation in intracerebral hemorrhage [10]. In neurocritical care, frequent QP detects delayed cerebral ischemia after aneurysmal subarachnoid hemorrhage [11]. In severe traumatic brain injury, QP trends mirror intracranial pressure dynamics and track outcomes [12]. QP also objectively documents absent brainstem reflexes in brain death evaluations [13]. After cardiac arrest, guideline‑recommended automated pupillometry supports prognostication [14].

Beyond traditional neurology settings, QP adoption now spans emergency departments (ED), operating rooms (OR), non-neurological intensive care units (ICUs), and wards, signalling the growing need for reliable QP in increasingly diverse environments [7,8,15]. In pre‑hospital care in particular, early feasibility work suggests first responders can apply QP during out‑of‑hospital emergencies, enabling earlier triage, monitoring, and decision‑making before hospital arrival [15,16]. The use of smartphone platforms for deep learning-based stabilization in mobile pupillometry is gaining prominence, offering particular utility in pre-hospital clinical contexts where rapid and reliable assessment is critical [17].

This pilot work may pave the way for more widespread adoption of QP, leveraging specialized algorithms and the evolving hardware of smartphones (e.g., light sensors). We demonstrate that computational pupillometry and the PuRe Score significantly advance neurological assessment and monitoring. Thus, advanced, lighting‑corrected methodologies, such as PuRe, represent a promising approach toward truly confounder‑free, non‑invasive neurological monitoring, emphasizing their potential clinical utility, including in non‑invasive ICP monitoring.

The limitations of this study include the single‑center design and relatively small sample size of a feasibility pilot. The study is not powered for granular subgroup analyses and broad generalizability. Larger and multicenter studies are needed to validate the findings across diagnoses, medication states, and care settings. Medication effects (e.g., propofol, fentanyl) may modulate pupillary dynamics; although medication presence is summarized at the recording level, dose-timing effects were not modeled. Pre‑existing ophthalmic conditions were not systematically assessed; future studies should include ophthalmologic screening to contextualize pupillary responses.

## Conclusions

Quantitative pupillometry augmented by the PuRe score leverages the imaging sensors and computing power of modern smartphones to potentially transform pupillary assessment - from a measure confounded by lighting into a reliable biomarker of neurological status. By correcting for ambient light and using high‑resolution imaging with AI‑driven analysis, the PuRe method can align the portability and affordability of consumer devices with the accuracy and reliability expected of specialized medical equipment.

Our findings suggest that such systems can reliably discriminate severe neurological impairment and track dynamic changes in pupil function across a spectrum of clinical environments. Standardizing the pupillary light reflex in this way may therefore improve prognostication of critical illness, support triage and treatment decisions outside the hospital, and enable more consistent neurological examinations across providers and care settings.

Future work should explore real-time integration across stages of care delivery, validation in larger and more diverse populations, and incorporation of automated pupillometry into multi-modal predictive models for neurological outcomes. Comparative studies against pupillary activity indices from other commercially available systems should also be planned. Further research should focus on elucidating ICP-PuRe correlations across diverse settings and ambient light conditions.
